# Tunable diffraction-free array in nonlinear photonic crystal

**DOI:** 10.1038/srep40856

**Published:** 2017-01-18

**Authors:** Dongmei Liu, Dunzhao Wei, Yong Zhang, Zhenhua Chen, Rui Ni, Bo Yang, Xiaopeng Hu, Y. Q. Qin, S. N. Zhu, Min Xiao

**Affiliations:** 1 National Laboratory of Solid State Microstructures, College of Engineering and Applied Sciences, and School of Physics, Nanjing University, Nanjing 210093, China; 2 Department of Physics, University of Arkansas, Fayetteville, Arkansas 72701, USA; 3 School of Physics and Telecommunication Engineering, South China Normal University, Guangzhou 510006, China

## Abstract

Diffraction-free beams have attracted increasing research interests because of their unique performances and broad applications in various fields. Although many methods have been developed to produce such beams, it is still challenging to realize a tunable non-diffracting beam. Here, we report the generation of a tunable diffraction-free array through second-harmonic generation in a nonlinear photonic crystal, i.e., a 2D periodically-poled LiTaO_3_ crystal. In such a crystal, the second-harmonic wave is engineered by properly designing the domain structure based on the Huygens-Fresnel principle. The characteristics of the generated diffraction-free array including its period, propagation length, and wavelength can be tuned by simply changing the input wavelength. Our observation not only enriches the diffraction-free optics, but also has potential applications for photolithography and imaging.

Diffraction, originating from the Helmholtz equation, has long been considered as a universal characteristic of all classical waves. However, Durnin *et al*. reported in 1987 an exact diffraction-free mode solution of the Helmholtz equation^1^,^2^, which has a transverse intensity distribution independent of the propagation distance. The first experimental demonstration was a nearly non-diffracting Bessel beam^1^. Since then, investigations of such diffraction-free beams and their applications in metrology^3^, nonlinear optics^4^, atomic optics^5^, optical micro-manipulation^6^,^7^,^8^, medical imaging^9^, electron microscope^10^, and wireless optical communications^11^ have become an active research area. Besides this Bessel beam, other non-diffracting solutions^12^,^13^ including the Airy beam^14^,^15^ were also discovered. So far, the diffraction-free beams are mainly generated through linear optical methods, such as Fabry-Pérot interferometer^16^, spatial light modulator^17^, holographic process^18^, diffractive phase elements^19^, axicon^20^ and surface plasmon polariton (SPP)^21^,^22^. The experiments using nonlinear optical techniques are less reported^23^,^24^,^25^. In most of these methods, the performances of the generated diffraction-free beam, such as its wavelength, beam size, and propagating length, are fixed in the devices. The few tunable methods require certain complicated instruments like spatial light modulators^17^. In this Letter, we propose and demonstrate a novel nonlinear optical method to produce a tunable diffraction-free array of beams in a single nonlinear photonic crystal, i.e., a two-dimensional (2D) periodically-poled LiTaO_3_ (PPLT) crystal.

PPLT crystals have been extensively investigated because they can realize highly-efficient frequency conversions through the quasi-phase-matching (QPM) technique^26^. Since the concept of nonlinear photonic crystal, i.e., 2D PPLT crystal, was proposed by Berger^27^ in 1998, numerous interesting phenomena have been discovered such as non-collinear second-harmonic generation (SHG)^28^, nonlinear Čerenkov radiations^29^,^30^,^31^, and nonlinear Talbot self-imaging^32^. Recently, domain engineering in nonlinear photonic crystals for spatial light modulation attracts an increasing research interest. Scientists have developed various domain structures to realize dual-focused second-harmonic (SH) spots^33^, conical SHG^34^,^35^, optical orbital angular momentum states^36^,^37^, beam shaping^38^,^39^, and superfocusing^40^. By utilizing the domain-engineering method based on the Huygens-Fresnel principle, we can design the desired domain structure to realize certain tunable diffraction-free SH array in a single PPLT chip. Our results not only extend the concept of diffraction-free optics, but also open a door for broader applications of non-diffracting beams in photolithography and imaging.

## Results

### Theory

**Figure 1:**
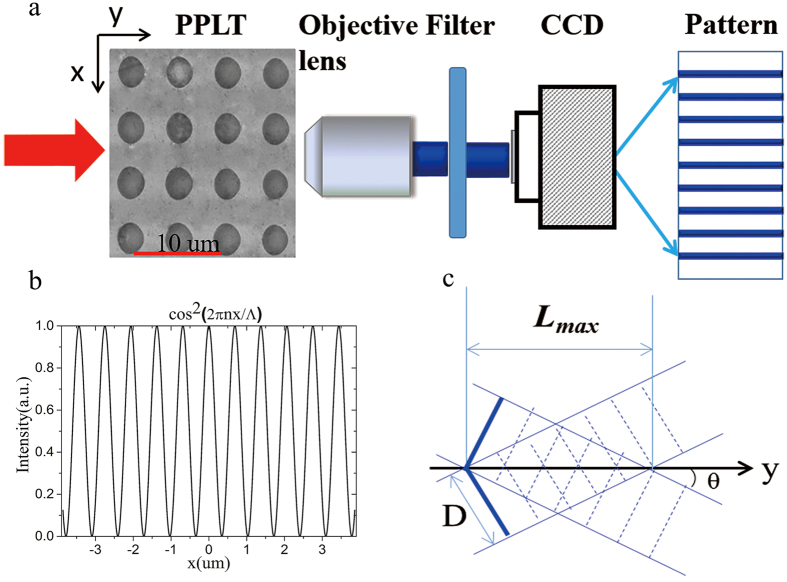
Experimental configuration. The experimental setup is shown in (**a**). The input laser propagates along the y-axis of the squarely-poled LiTaO_3_ crystal. The generated SH pattern is recorded by a CCD camera. The diffraction-free cosine beam in (**b**) can be decomposed into two components (**c**).

The idea is intrigued by the free-space Bessel beam, in which the diffraction-free field can be decomposed into plane-wave components with wave vectors on a cone^1^. Similarly, two plane waves can form a diffraction-free array with a cosine transverse profile, which has been demonstrated in SPPs^21^. Such cosine beam can be considered as the 2D counterpart of the Bessel beam^21^. Here, we produce a tunable diffraction-free array through a SHG process in a 2D PPLT crystal as shown in Fig. 1a. The coupled-wave equation can be written as


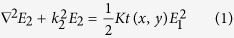


where *E*_1_ and *E*_2_ are the electrical fields of the fundamental wave and SH wave, respectively; *k*_2_ is the wave vector of the SH wave; *K* is the coupling coefficient; and *t*(*x*, *y*) is the structural function of the PPLT crystal. We seek a diffraction-free cosine solution for the SH wave (Fig. 1b), which can be written as


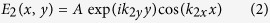


Here, A is a constant, *k*_2x_ and *k*_2y_ are the x and y components of the SH wave vectors, respectively. The electrical field distribution along z direction is uniform in this case. The cosine beam described by Eq. (2) can be decomposed into two plane-wave components (Fig. 1c). Obviously, such solution has a transverse intensity profile independent of the propagation direction y, which represents a diffraction-free SH array. Based on Eqs (1) and (2), we use the nonlinear Huygens-Fresnel principle^33^ to engineer the domain structure *t*(*x*, *y*) for the realization of such beam (see Methods for the details). As well known, it is impossible to experimentally realize an ideal Bessel beam because it carries infinite energy^1^. Alternatively, one can generate a Gaussian-Bessel beam (i.e. the Bessel solution modulated by a Gaussian envelope) in experiment, which preserves the diffraction-free properties in the paraxial approximation^41^. Because the non-diffracting solution in Eq. (2) suffers from the same problem, we introduce the cosine-Gaussian beam^21^ in the experiment.

**Figure 2:**
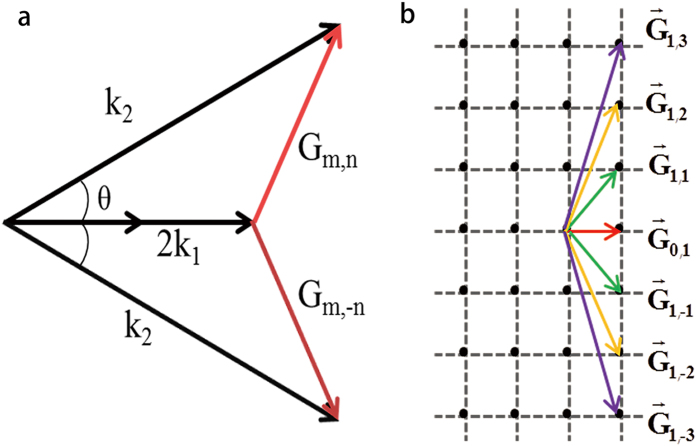
Noncollinear QPM. The diffraction-free cosine beam can be understood from the non-collinear QPM configuration (**a**). The reciprocal vectors *G*_*m,n*_ and *G*_*m*,−*n*_ can produce two non-collinear SH waves, which can be considered as the decomposed components of the cosine beam. The reciprocal vectors in a squarely-poled PPLT crystal are shown in (**b**).

The cosine beam consisting of two SH components can be understood through another view point, i.e., QPM. In a typical QPM configuration, SHG can be greatly enhanced by using the reciprocal vectors in a PPLT crystal to compensate for the phase mismatch between the fundamental wave and the SH wave^26^. Luckily, there exist abundant non-collinear reciprocal vectors in a 2D domain structure, which can realize non-collinear QPM SHG^27^,^28^ as shown in Fig. 2a. For instance, in a squarely-poled LiTaO_3_ crystal as shown in Fig. 1a, the reciprocal vectors (Fig. 2b) are defined by


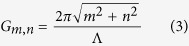


where the subscripts *m* and *n* denote the orders of the reciprocal vector along the longitudinal and transverse directions, respectively. 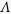 is the period of the domain structure. The QPM condition under non-collinear configuration (Fig. 2a) requires


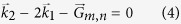


where *k*_1_ and *k*_2_ are the wave vectors of the fundamental and SH waves, respectively. Interestingly, G_m,n_ and its mirror-symmetrical vector G_m,−n_ can simultaneously generate two SH waves as shown in Fig. 2a, which can be considered as the decomposed components of the cosine beam in Eq. (2). They interfere with each other and result in a diffraction-free SH array as shown in Fig. 1b and c. Considering that the two SH components of the cosine beam have an in-between angle of 2θ (decided by Equation (4)), the transverse profile of the SH intensity (Fig. 1b) can be easily deduced to be


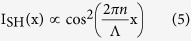


Obviously, the period of the obtained SH array is


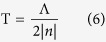


From Fig. 1c, the propagation distance of the diffraction-free beam can be written as


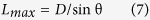


where *D* is the diameter of the SH component. Unlike other diffraction-free schemes which are usually fixed by the sample structures, a given PPLT structure can actually generate diffraction-free arrays with varied periods and propagation distances by involving different G_m,n_. This can be easily realized by changing the input wavelength or tuning the operation temperature. In our scheme, the prerequisite to realize diffraction-free is to satisfy the non-collinear QPM condition. If not fulfilled, one cannot obtain the diffraction-free array because no non-collinear SH beams are efficiently generated.

### Experimental demonstration of the tunable diffraction-free array

The PPLT crystal for the generation of a diffraction-free SH array is designed to have a squarely-poled structure with a period of Λ = 5.5 μm (Fig. 1a). A Ti:Sapphire femtosecond laser serves as the input fundamental field, which can be continuously tuned from 690 nm to 1050 nm in wavelength. This fundamental beam is first reshaped to produce a near-parallel beam. Then, it travels along the y direction with its polarization parallel to the z-axis of the crystal (Fig. 1a). The coordinate system is set according to the crystal axis. Under this experimental configuration, the involved nonlinear optical coefficient d_33_ is the biggest one in the LiTaO_3_ crystal. A short-pass filter is placed after the crystal to block the fundamental field. The SH patterns near the PPLT crystal are magnified by a 100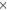 objective lens with a N.A. = 0.7 and then recorded by a CCD camera. By moving the objective lens along the y direction, we can investigate the diffractive characteristics of the SH patterns.

**Figure 3:**
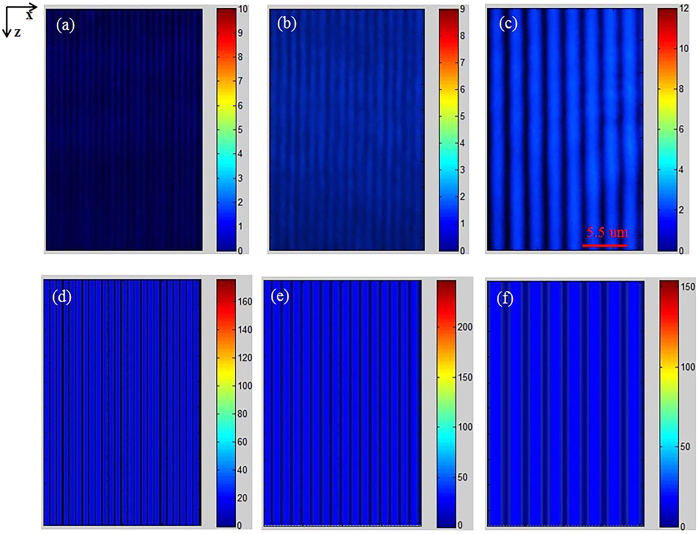
Diffraction-free SH arrays generated by different fundamental wavelengths. The measured (**a**–**c**) and simulated (**d**–**f**) cross sections of the diffraction-free SH arrays at certain observation planes. The periods of the array in the experiment are 0.92 μm, 1.38 μm, and 2.75 μm at the fundamental wavelengths of 906 nm (**a**), 928 nm (**b**) and 944 nm (**c**), respectively, which are well consistent with the corresponding numerical simulations.

**Figure 4:**
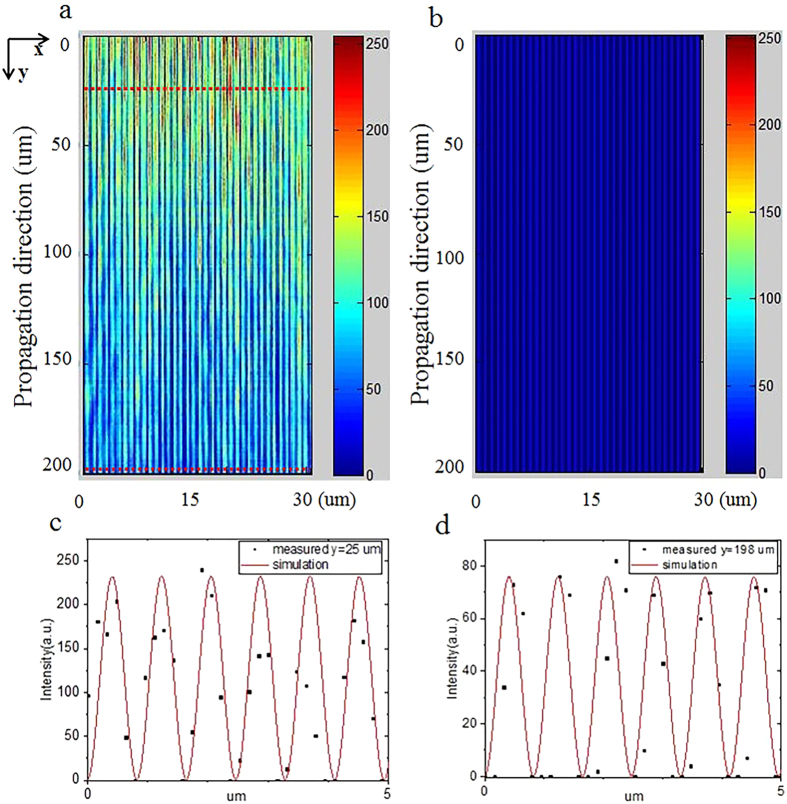
Diffraction-free “carpets”. Experimental (**a**) and theoretical (**b**) diffraction-free “carpets” along the propagation direction are obtained at a 906 nm input laser. (**c**) and (**d**) show the deviations of the measured beam profile from an ideal non-diffracting array at propagation distances of 25 μm and 198 μm, respectively.

The input laser is first set to be 906 nm. At this wavelength, the non-collinear SHG can be phase-matched with the reciprocal vectors G_1,3_ and G_1,−3_ (Fig. 2). At the output face of the PPLT crystal, one can observe a SH array with a period of 0.92 μm as shown in Fig. 3a, which is well consistent with the theoretical period of 0.917 μm from Eq. (6) with |n| = 3. The corresponding numerical simulation based on the Huygens-Fresnel principle is shown in Fig. 3d. The period of the simulated SH pattern is 0.93 μm. The small deviation may result from that the dispersion relation of the LiTaO_3_ crystal used in the calculation does not perfectly match our sample. By moving the objective lens along the y direction, we can record the SH patterns at different observation planes. Figure 4a shows the measured evolution of the SH carpet within y = 200 μm, which clearly presents the diffraction-free performance. As propagating along the y direction, the intensity of the SH array decreases because of the cosine-Gaussian mode; however, the array period does not change. The numerical simulation in Fig. 4b also confirms such diffraction-free behavior. For simplicity, we have assumed a plane-wave illumination in simulations, which cannot predict the attenuation of the SH intensity along the propagation direction in Fig. 4a. As shown in Fig. 4c and d, the standard deviation from the theoretical diffraction-free array increases from 1.37 × 10^−4^ to 2.29 × 10^−4^ as the experimentally generated cosine-Gaussian beam propagates from y = 25 μm to y = 198 μm. It should be noted that the SH array presents such diffraction-free performance only near the center of the whole picture (within the area of ~100 μm × 10  μm in our experiment) because of the Gaussian modulation of the Bessel solution.

Next, we change the input laser wavelength to 928 nm and 944 nm, respectively, to tune the diffraction-free array. The fundamental beam power is kept at 50 mW for all the wavelengths. As shown in Fig. 3b and c, the SH arrays change dramatically comparing to the pattern excited by a 906 nm fundamental beam (Fig. 3a). The period of the SH pattern at the pump wavelength of λ = 928 nm is 1.38 μm (Fig. 3b), which is one quarter of the domain period. When further increasing the input wavelength to λ = 944 nm, the array period becomes 2.75 μm (Fig. 3c). The dependence of the period of the diffraction-free array on the wavelength originates from the involvement of different reciprocal vectors, and therefore G_1,2_/G_1,−2_ (Fig. 2) at 928 nm and G_1,1_/G_1,−1_ (Fig. 2) at 944 nm correspond to 1/4 and 1/2 of the domain period, respectively, according to Eq. (6). The numerical simulations based on Huygens-Fresnel principle for these two cases are shown in Fig. 3e and f, which are well in agreement with the experimental results. Our measurement shows that the non-diffracting SH pattern can be observed at a distance of up to 4.5 mm away from the sample with an input wavelength of 944 nm. This is slightly shorter than the theoretically predicted propagation length of 4.8 mm from Eq. (7) with D = 400 μm. From the experimental images (Fig. 3a–c), one can easily see that the intensity of the SH pattern increases when the laser is tuned to a longer wavelength, which is mainly caused by the more effective nonlinear coefficient. Usually, a lower-order G_m,n_ has a higher effective nonlinear coefficient, which can realize a higher SHG efficiency. At a non-QPM wavelength, we can hardly observe a diffraction-free array pattern in the experiment because the prerequisite condition has been broken.

In principle, for each pair of G_m,n_ and G_m,−n_ in a 2D PPLT crystal, one can always find a suitable wavelength to satisfy the non-collinear QPM condition and then to generate the diffraction-free array. However, this might not be realizable in experiment because (1) the high-order reciprocal vector may have an effective nonlinear coefficient which is too small to efficiently generate the SH waves; (2) the above prediction is only valid under the paraxial approximation, which rules out its applicability to the reciprocal vectors with big subscript *n*. To design a practical PPLT crystal for generating such non-diffracting arrays, it is important to suppress the collinear SHG process because the unwanted background could completely ruin the diffraction-free beam pattern. Usually, the input wavelength should be chosen as far as possible away from the QPM wavelength for the collinear SHG process.

## Discussion

In conclusion, we have presented the generation of a tunable diffraction-free array, i.e. cosine-Gaussian beam, through non-collinear QPM SHG processes in a nonlinear photonic crystal. Beyond the previously demonstrated techniques, this work has extended the generation of diffraction-free beam in two fronts. First, the introduction of SHG produces a non-diffraction array at a shorter wavelength, which can have potential applications in photolithography and optical imaging. The resolution of the beam is improved by a factor of 2 due to SHG. Second, one can easily tune several characteristics of the diffraction-free cosine-Gaussian beam in a single chip, which makes it more convenient to utilize it in integrated photonic devices. Here, we have demonstrated the wavelength- and range-tunable non-diffracting array in a PPLT crystal by using varied input wavelength. Actually, there are more tools to modulate and optimize the generated array in the PPLT crystals. For example, by utilizing the excellent thermo-optical, electro-optical and acoustic-optical performances of the LiTaO_3_ crystal, the diffraction-free array can be modulated by changing the operation temperature, and applying an electrical or acoustic field. The performance of the non-diffracting array can be further improved through additional domain engineering techniques. For instance, it has been experimentally shown that chirped, ring-shaped and quasi-periodic structures can greatly enhance the tunable properties of the QPM processes^42^,^43^. One can utilize these structures to further tune and modify the non-diffracting array. Most importantly, such nonlinear photonic crystals provide a useful integrated platform to manipulate the propagations of diffraction-free beam arrays and other spatial light beams for their potential applications in photolithography and optical imaging.

## Methods

### Analytical expressions of the fundamental field *E*_1_ and the SH field *E*_2_ for numerical simulations

The numerical simulations are performed by using the Huygens-Fresnel principle^33^, in which each part of the crystal is considered as a point source which emits the SH wave. There is a π phase-shift between the SH waves generated from positive and negative domains^44^. The input fundamental beam propagates along the y axis of the crystal (Fig. 1). Under the slowly-varying-envelope approximation, the evolutions of the fundamental field *E*_1_ and the SH field *E*_2_ in the squarely-poled LiTaO_3_ crystal can be described by


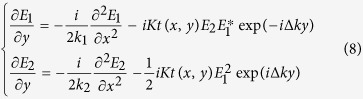


where 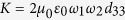 is the coupling coefficient with *d*_33_ being the nonlinear coefficient of the crystal. 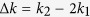 is the phase mismatch between the fundamental wave and the SH wave. From Eq. (8), one can write the difference equations as


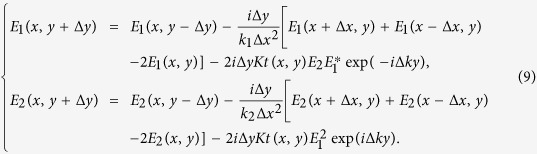


Here, Δ*x* and Δ*y* are the space steps. Then, we apply the finite difference method^45^ to calculate SHG process in the 2D PPLT crystal. It should be noted that the above equations are valid in the paraxial approximation.

## Additional Information

**How to cite this article:** Liu, D. *et al*. Tunable diffraction-free array in nonlinear photonic crystal. *Sci. Rep.* **7**, 40856; doi: 10.1038/srep40856 (2017).

**Publisher's note:** Springer Nature remains neutral with regard to jurisdictional claims in published maps and institutional affiliations.

## References

[b1] Durnin J. & Miceli J. J. Jr. Diffraction-free beams. Phys. Rev. Lett. 58, 1499–1501 (1987). 10034453 10.1103/PhysRevLett.58.1499

[b2] Durmin J. Exact solutions for nondiffracting beams. I. the scalar theory. J. Opt. Soc. Am. A 4, 651–654 (1987).

[b3] Arimoto R., Saloma. C., Tanaka. T. & Kawata S. Imaging properties of axicon in a scanning optical system. Appl. Opt. 31, 6653–6657 (1992). 20733892 10.1364/AO.31.006653

[b4] Wulle T. & Herminghaus S. Nonlinear optical of Bessel beams. Phys. Rev. Lett. 70, 1401–1404 (1993). 10053283 10.1103/PhysRevLett.70.1401

[b5] Arlt J., Dholakia K., Soneson J. & Wright E. M. Optical dipole traps and atomic waveguides based on Bessel light beams. Phys. Rev. A 63, 063602 (2001).

[b6] Arlt J. G. V., Sibbett W. & Dholakia K. Optical micromanipulation using a Bessel light beam. Opt. Commun. 197, 239–245 (2001).

[b7] Garces-Chavez V., McGloin D., Melville H., Sibbett W. & Dholakia K. Simultaneous micromanipulation in multiple planes using a self-reconstructing light beam. Nature 419, 145–147 (2002). 12226659 10.1038/nature01007

[b8] McGloin D., Garces-Chavez V. & Dholakia K. Interfering Bessel beams for optical micromanipulation. Opt. Lett. 28, 657–659 (2003). 12703932 10.1364/ol.28.000657

[b9] Saari P. & Reivelt K. Evidence of x-shaped propagation-invariant localized light waves. Phys. Rev. Lett. 79, 4135–4138 (1997).

[b10] Grillo V. . Generation of nondiffracting electron Bessel beams. Phys. Rev. X 4, 011013 (2014).

[b11] Kollarova V. . Application of nondiffracting beams to wireless optical communications. SPIE Proceedings 3736 (2007).

[b12] Gutiérrez-Vega J. C., Iturbe-Castillo M. D. & Chávez-Cerda. S. Alternative formulation for invariant optical fields: Mathieu beams. Opt. Lett. 25, 1493–1495 (2000). 18066256 10.1364/ol.25.001493

[b13] Bandres M. A., Gutiérrez-Vega J. C. & Chávez-Cerda S. Parabolic nondiffracting optical wave fields. Opt. Lett. 29, 44–46 (2004). 14719655 10.1364/ol.29.000044

[b14] Siviloglou G. A., Broky J., Dogariu A. & Christodoulides D. N. Observation of accelerating Airy beams. Phys. Rev. Lett. 99, 213901 (2007). 18233219 10.1103/PhysRevLett.99.213901

[b15] Kaminer I., Bekenstein R., Nemirovsky J. & Segev M. Nondiffracting accelerating wave packets of Maxwell’s equations. Phys. Rev. Lett. 108, 163901 (2012). 22680719 10.1103/PhysRevLett.108.163901

[b16] Cox A. J. & Dibble D. C. Nondiffracting beam from a spatially filtered Febry-Perot resonator. J. Opt. Soc. Am. A 9, 282–286 (1992).

[b17] Davis J. A., Guertin J. & Cottrell D. M. Diffraction-free beams generated with programmable spatial light modulators. Appl. Opt. 32, 6368–6370 (1993). 20856473 10.1364/AO.32.006368

[b18] Vasara A., Turunen J. & Friberg A. T. Realization of general nondiffracting beams with computer-generated holograms. J. Opt. Sco. Am. A 6, 1748–1754 (1989). 10.1364/josaa.6.001748 2585173

[b19] Cong W. X., Chen N. X. & Gu B. Y. Generation of nondiffracting beams by diffractive phase elements. J. Opt. Sco. Am. A 15, 2362–2364 (1998).

[b20] Weber N., Spether D., Seifert A. & Zappe H. Highly compact imaging using Bessel beams generated by ultraminiaturized multi-micro-axicon systems. J. Opt. Soc. Am. A 29, 808–816 (2012). 10.1364/JOSAA.29.000808 22561940

[b21] Lin J. . Cosine-Gauss plasmon beam: a localized long-range nondiffracting surface wave. Phys. Rev. Lett. 109, 093904 (2012). 23002838 10.1103/PhysRevLett.109.093904

[b22] Li L., Li T., Wang S. M. & Zhu S. N. Collimated plasmon beam: nondiffractiing versus linearly focused. Phys. Rev. Lett. 110, 046807 (2013). 25166192 10.1103/PhysRevLett.110.046807

[b23] Saltiel S., Krolikowshi W., Neshev D. & Kivshar Y. S. Generation of Bessel beams by parametric frequency doubling in annular nonlinear periodic structures. Opt. Exp. 15, 4132–4138 (2007). 10.1364/oe.15.004132 19532656

[b24] Ellenbogen T., Voloch-Bloch N., Ganany-Padowicz A. & Arie A. Nonlinear generation and manipulation of airy beams. Nature Photon. 3, 395–398 (2009).

[b25] Dolev I. & Aire A. Three wave mixing of airy beams in a quadratic nonlinear photonic crystals. Appl. Phys. Lett. 97, 171102 (2010).

[b26] Armstrong J. A., Bloembergen N., Ducuing J. & Pershan P. S. Interaction between light waves in a nonlinear dielectric. Phys. Rev. 127, 1918–1939 (1962).

[b27] Berger V. Nonlinear photonic crystals. Phys. Rev. Lett. 81, 4136–4139 (1998).

[b28] Moscovich S. . Noncollinear second-harmonic generation in sub-micrometer-poled RbTiOPO_4_. Opt. Express. 12, 2236–2242 (2004). 19475059 10.1364/opex.12.002236

[b29] Sheng Y. . Čerenkov-type second-harmonic generation with fundamental beams of different polarizations. Opt. Lett. 35, 1317–1319 (2010). 20436554 10.1364/OL.35.001317

[b30] Ren H. J., Deng X. W., Zheng Y. L., An N. & Chen X. F. Nonlinear Čerenkov radiation in an anomalous dispersive medium. Phys. Rev. Lett. 108, 223901 (2012). 23003594 10.1103/PhysRevLett.108.223901

[b31] Saltiel S. M. . Cerenkov-type second-harmonic generation in two-dimensional nonlinear photonic structures. IEEE J. Quantum Elect. 45, 1465–1472 (2009).

[b32] Zhang Y., Wen J. M., Zhu S. N. & Xiao M. Nonlinear Talbot effect. Phys. Rev. Lett. 104, 183901 (2010). 20482176 10.1103/PhysRevLett.104.183901

[b33] Qin Y. Q., Zhang C., Zhu Y. Y., Hu X. P. & Zhao G. Wave-front engineering by Huygens-Fresnel principle for nonlinear optical interactions in domain engineered structures. Phys. Rev. Lett. 100, 063902 (2008). 18352473 10.1103/PhysRevLett.100.063902

[b34] Xu P. . Conical second harmonic generation in a two-dimensional χ^(2)^ photonic crystal: a hexagonally poled LiTaO_3_ crystal. Phys. Rev. Lett. 93, 133904 (2004). 15524722 10.1103/PhysRevLett.93.133904

[b35] Saltiel S. M. . Generation of second-harmonic conical waves via nonlinear Bragg diffraction. Phys. Rev. Lett. 100, 103902 (2008). 18352187 10.1103/PhysRevLett.100.103902

[b36] Li S. M. . Managing orbital angular momentum in second-harmonic generation. Phys. Rev. A 88, 035801 (2013).

[b37] Fang X. Y. . orbital angular momentum states through second-harminc generation in a two-dimensional periodically poled LiTaO_3_ crystal. Appl. Phys. Lett. 107, 161102 (2015).

[b38] Shapira A., Shiloh R., Juwiler I. & Arie A. Two-dimensional nonlinear beam shaping. Opt. Lett. 37, 2136–2138 (2012). 22660146 10.1364/OL.37.002136

[b39] Shay K. Z., Avayu O., Michaeli L. & Ellenbogen T. Nonlinear beam shaping with Plasmonic Metasurfaces. ACS Photonics 3, 117–123 (2016).

[b40] Liu D. M. . Diffraction interference induced superfocusing in nonlinear Talbot effect. Sci. Rep. 4, 6134 (2014). 25138077 10.1038/srep06134 PMC4138516

[b41] Gori F., Guattari G. & Padovani C. Bessel-Gauss beams. Opt. Commun. 64, 491–495 (1987).

[b42] Arbore M. A., Galvanaushas A., Harter D., Chou M. H. & Fejer M. M. Engineerable compression of ultrashort pulses by use of second-harmonic generation in chirped-period-poled lithium niobate. Opt. Lett. 22, 1341–1343 (1997). 18188233 10.1364/ol.22.001341

[b43] Zhang C. . Third-harmonic generation in a general two-component quasi-periodic optical superlattice. Opt. Lett. 26, 899–901 (2001). 18040485 10.1364/ol.26.000899

[b44] Wei D. Z., Liu D. M., Hu X. P., Zhang Y. & Xiao M. Superposed second-harmonic Talbot self-image from a PPLT crystal. Laser Phys. Lett. 11, 095402 (2014).

[b45] Zhou M. S., Ma J. C., Zhang C. & Qin Y. Q. Numerical simulation of nonlinear field distributions in two-dimensional optical superlattices. Opt. Exp. 20, 1261–1267 (2012). 10.1364/OE.20.001261 22274471

